# Comparative Outcomes of Different Surgical Approaches for Non‐Lactational Mastitis With Posterior and Non‐Posterior Space Abscesses: A Retrospective Cohort Study

**DOI:** 10.1002/iid3.70291

**Published:** 2025-10-23

**Authors:** WenJie Zhang, Hailiang Ren, Jian Wu

**Affiliations:** ^1^ The Center of Breast and Thyroid Surgery, Department of General Surgery The Third People's Hospital of Chengdu & The Affiliated Hospital of Southwest Jiaotong University Chengdu China; ^2^ Department of Gastrointestinal, Bariatric and Metabolic Surgery, and Research Center for Nutrition, Metabolism and Food Safety, West China‐PUMC CC.C. Chen Institute of Health, West China School of Public Health and West China Fourth Hospital Sichuan University Chengdu China

**Keywords:** abscess, non‐lactation mastitis, operation

## Abstract

**Objective:**

This study aims to compare the outcomes of different surgical approaches for treating non‐lactational mastitis, with a particular focus on the differences between posterior and non‐posterior space abscesses. It evaluates the effectiveness of Postoperative Vacuum Drainage (POVD) in enhancing recovery and alleviating pain compared to traditional methods.

**Methods:**

A retrospective analysis was conducted on 98 female patients diagnosed with non‐lactational mastitis at the Third People's Hospital of Chengdu between March 2014 and December 2020. Patients were classified into posterior space abscess (*n* = 40) and non‐posterior space abscess (*n* = 58) groups. Clinical data, treatment modalities, and outcomes were compared. Statistical analyses included the Independent *t*‐test for continuous variables and the chi‐square test for categorical variables, with significance set at *p* < 0.05.

**Results:**

Patients with posterior space abscesses exhibited higher preoperative Visual Analog Scale (VAS) scores (7 [IQR: 5–8] and 5 [IQR: 3–6], *p* < 0.05) and more severe pain. Their complete response time was also longer (6.0 vs. 5.0 months, *p* < 0.05). The use of incision and drainage decreased by 69.4%, while percutaneous aspiration increased annually. For posterior space abscesses, combined operations (incision, drainage, and POVD) significantly shortened response time, reduced the number of punctures, and improved postoperative VAS scores compared to percutaneous aspiration alone (*p* < 0.05).

**Conclusion:**

Posterior space abscesses were associated with more severe symptoms and prolonged healing. Combined treatments incorporating POVD demonstrated superior effectiveness over single percutaneous aspiration. POVD is highlighted as a promising, minimally invasive, and cost‐effective solution for managing non‐lactational mastitis with posterior space abscesses.

## Graphical abstract

Comparative Outcomes of Different Surgical Approaches for Non‐Lactational Mastitis with Posterior and Non‐Posterior Space Abscesses: A Retrospective Case‐Control Study on the Innovative Use of Vacuum‐Assisted Needle Drainage.

## 1 Introduction

Non‐lactational mastitis (NLM) refers to a group of nonspecific inflammatory diseases with unknown causes that predominantly affect women during non‐breastfeeding periods [1]. Pathological subtypes include mammary duct ectasia (MDE)/periductal mastitis (PDM) and granulomatous lobular mastitis. Patients often present with a firm breast lump that mimics carcinoma in the early stages, while abscesses typically develop later, often in central or subareolar locations. These abscesses are reported to have a distinct microbial spectrum compared to lactational abscesses and are usually accompanied by varying degrees of pain [2]. Although the exact etiology remains unclear, it is thought to result from a complex interplay of infectious agents, immune dysregulation, and possibly genetic factors. Early or mild cases are often managed conservatively with antibiotics, pain relief, and anti‐inflammatory medications to reduce inflammation and control infection. However, recurrent or severe abscesses may require surgical intervention. Unlike acute inflammation seen in common infections, NLM is characterized by chronic inflammation, which can lead to granuloma formation and significant tissue damage.

The immune response plays a critical role in the progression and severity of NLM [3]. Chronic inflammation in NLM is often triggered by an initial infection or an autoimmune response, wherein the immune system mistakenly targets healthy breast tissue [4, 5]. This leads to persistent inflammation and abscess formation. For example, granulomatous lobular mastitis is marked by granulomas—clusters of immune cells attempting to contain the inflammation [6]. This chronic inflammatory state suggests that repeated surgical interventions may exacerbate rather than alleviate the condition, highlighting the challenges in managing NLM effectively.

Conventional surgical treatments, such as incision and drainage, may provoke stronger inflammatory responses due to physical trauma and the potential introduction of new pathogens [7, 8]. This can create a cycle of inflammation and tissue damage, further worsening the condition. As a result, less invasive management strategies, including percutaneous needle aspiration under ultrasound guidance [9], ductal lavage [10], and vacuum‐assisted needle drainage (VAND) [11, 12], are increasingly being explored [13, 14]. These methods aim to minimize physical trauma, thereby reducing additional inflammatory responses.

The study of NLM provides critical insights into its unique pathophysiology and effective treatment strategies. Chronic inflammation and immune response abnormalities in NLM can lead to granuloma formation and significant tissue damage. Understanding these mechanisms and developing effective treatments contribute to the broader understanding of inflammatory breast diseases. This underscores the importance of minimally invasive techniques like Postoperative Vacuum Drainage (POVD). Tailored treatment approaches based on abscess location are essential to improve patient outcomes and reduce recurrence rates [15].

Our findings highlight the potential of innovative, minimally invasive treatment options, such as POVD, to improve outcomes for patients with NLM by reducing pain, inflammation, and recurrence risk. By exploring the role of POVD in NLM treatment, this study offers a promising alternative to traditional surgical methods. It advances the understanding of both surgical and nonsurgical interventions for chronic inflammatory breast conditions, providing a foundation for future research and clinical improvements. This study advocates for more effective, patient‐centered treatment strategies.

## 2 Method

### 2.1 Data Collection

In this retrospective cohort study, we systematically analyzed patients diagnosed with non‐lactational mastitis with abscesses between March 2014 and December 2020 at our hospital, divided into posterior and non‐posterior abscess groups. This analysis was approved by the Medical Ethics Committee of the Third People's Hospital of Chengdu (Ethical Approval Number: 2021‐S‐75).

**Figure 1:**
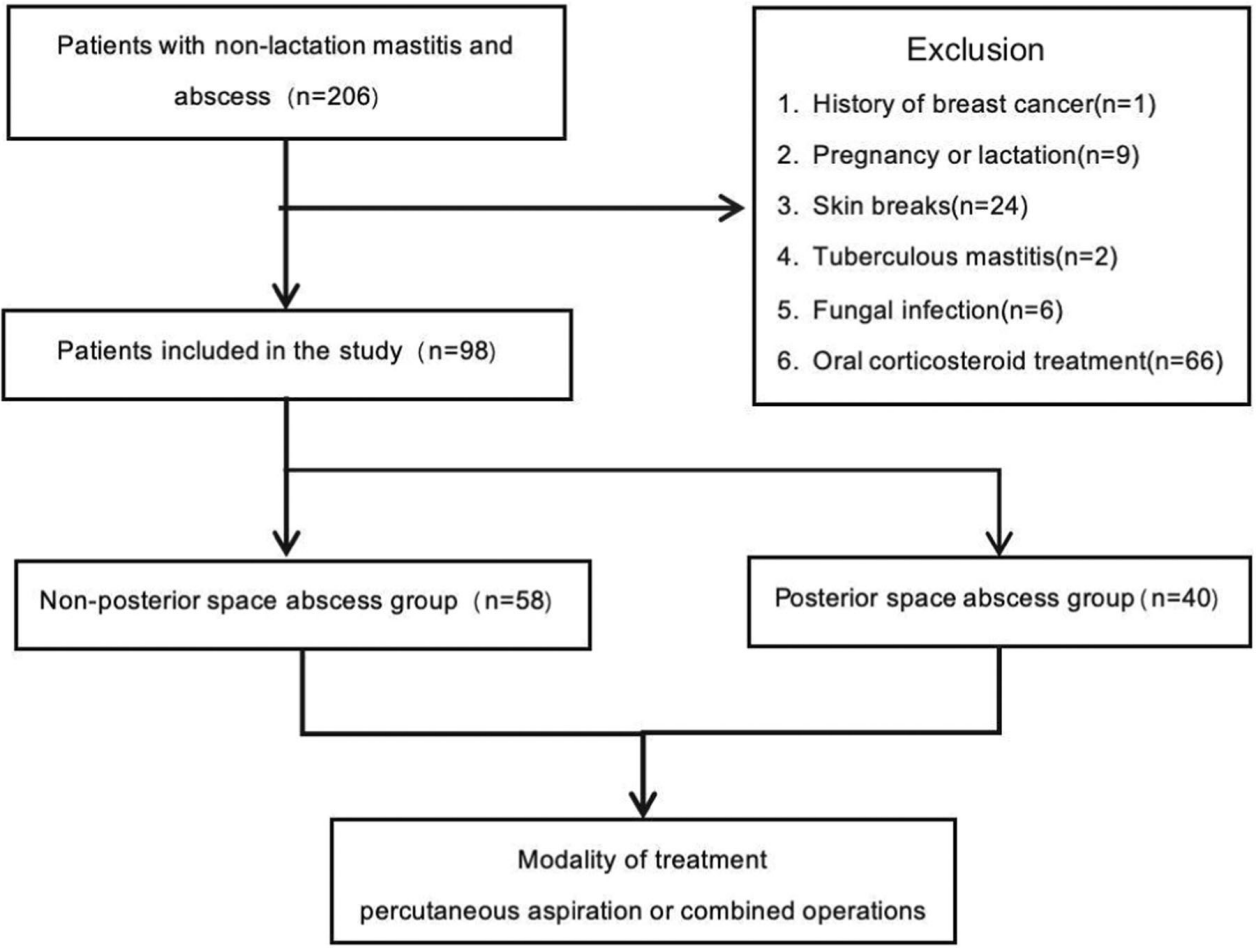
Patient inclusion flow chart. Flow‐chart showing the details of the inclusion process of patients for the study.

Patients were excluded if they met any of the following criteria: (1) history of breast cancer, due to the altered anatomy and physiology of the breast tissue, which could significantly influence both the development and treatment of non‐lactational mastitis; (2) pregnancy or lactation, as the hormonal and structural changes in breast tissue during these periods could confound the results by drastically altering the breast's response to infections and interventions; (3) presence of skin breaks; (4) cancerous mastitis; (5) fungal infections; and (6) use of oral corticosteroids. By excluding these groups, we aimed to maintain a homogeneous patient population, thereby enhancing the validity and applicability of the findings to non‐lactational mastitis patients (Figure 1).

In this study, “posterior space abscesses” are defined as those located in the deeper layers of breast tissue, closer to the chest wall, specifically within less than 2 cm from the pectoral muscles, as precisely evaluated using ultrasound imaging. In contrast, “non‐posterior space abscesses” are situated in the more superficial layers, which are generally more accessible for treatments such as aspiration or incision. This distinction was made to address the clinical challenges posed by the deeper location and severity of posterior space abscesses, which often require more advanced interventions. By classifying abscesses based on their anatomical location, we aimed to develop tailored treatment strategies that optimize patient outcomes. This classification is motivated by the need to tailor treatment strategies based on the anatomical complexity and clinical severity of the abscesses. Posterior space abscesses are often more challenging to access due to their deeper location and proximity to vital structures, requiring more advanced techniques such as incision, drainage, and postoperative vacuum drainage (POVD). In contrast, non‐posterior space abscesses, being superficial, are generally manageable with less invasive methods like percutaneous aspiration.

During the ultrasound assessment, a trained radiologist measured the distance from the skin surface to both the nearest edge of the abscess and the chest wall. This methodology not only ensured a consistent and reproducible approach to classifying the abscess locations but also facilitated the development of location‐specific treatment protocols. By differentiating these two types of abscesses, we aim to optimize patient outcomes by selecting the most appropriate and effective intervention for each type, reflecting the first attempt in the literature to address the significance of abscess location in treatment planning.

### 2.2 Procedure

Percutaneous aspiration:The size of the abscess was measured using ultrasound, and patients underwent percutaneous aspiration under local anesthesia. The puncture site was selected based on the location of the abscess, typically guided by the fluctuation of the breast tissue or the lower edge of the abscess. Under ultrasound guidance, a fine needle was inserted into the center and bottom of the abscess for drainage.

**Figure 2:**
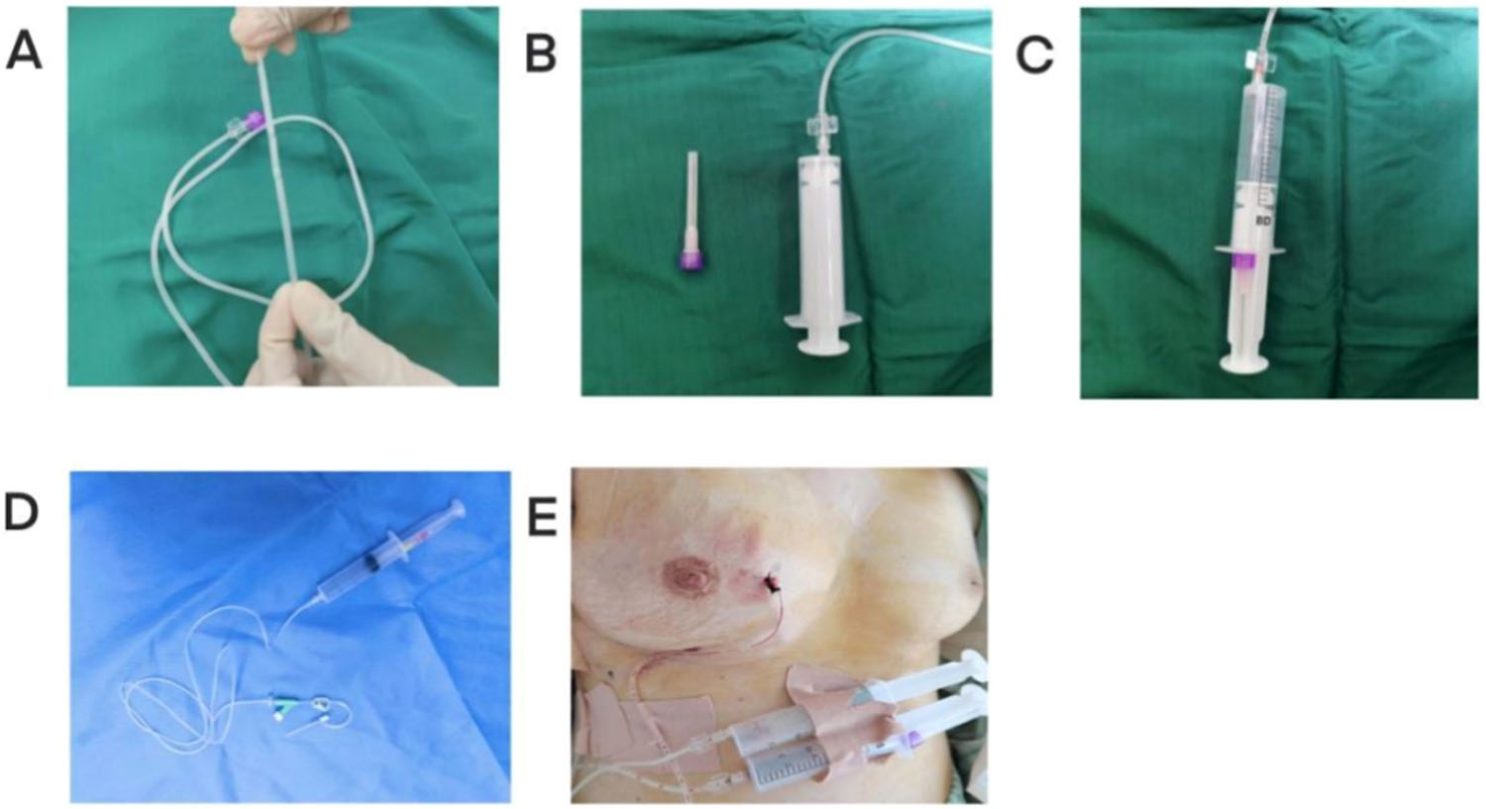
(A) The infusion extension tube with lateral holes at the tip for enhanced drainage and irrigation; (B) The infusion extension tube connected to a hypodermic syringe, ready for the creation of negative pressure; (C) The hypodermic syringe after creating negative pressure, ready for application in the vacuum‐assisted needle drainage (POVD) system; (D) The indwelling needle connected to the infusion extension tube, which is then linked to POVD, typically used for managing shallower abscesses; (E) The postoperative application of the POVD system, either with the infusion extension tube or indwelling needle in the abscess cavity, enabling continuous negative pressure drainage.

**Figure 3:**
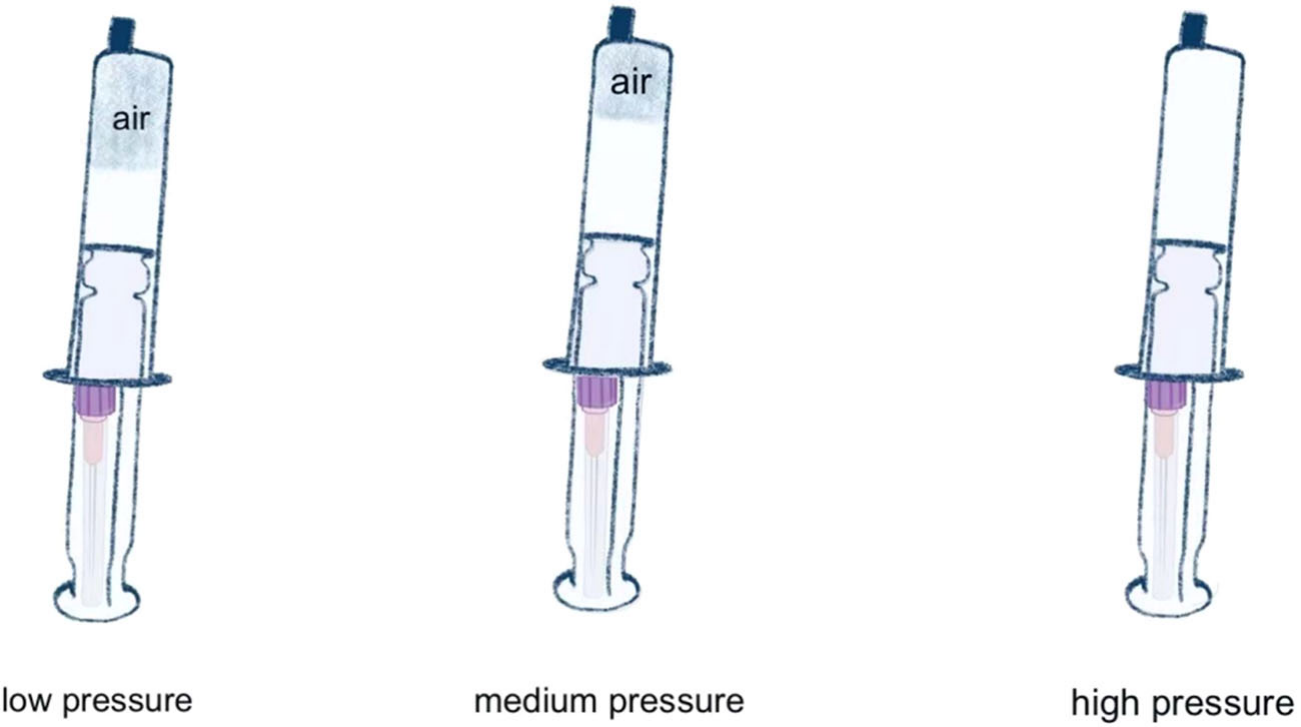
Controlling Suction Pressure Using Different Syringe Sizes in Vacuum‐Assisted Needle Drainage (POVD).

Combined Operations:A two‐step combined operation was employed, focusing on the innovative Postoperative Vacuum Drainage (POVD) technique (Figure 2).

a. Incision and Drainage:
  Initially, the abscess size was assessed using ultrasound. Under general anesthesia, a small incision (approximately 1‐2 cm) was made at the infra‐mammary border or over the lower portion of the abscess's projection on the body surface. For septated abscesses, forceps were used to open the septa and facilitate proper drainage.
b. Postoperative Vacuum Drainage (POVD):
  After incision and drainage, the POVD procedure was initiated. The POVD system was used in two different forms, depending on the abscess location and characteristics:

For posterior space abscesses: An infusion extension tube was carefully nserted into the abscess cavity. This tube was connected to a syringe, creating a vacuum for effective drainage. A three‐way valve was attached to the tube, allowing for continuous flushing and draining of the abscess cavity. The negative pressure strength was adjustable depending on the volume of air within the cavity, with syringes of varying sizes (5 ml, 10 ml, 20 ml, or 50 ml) used, as shown in Figure 3. This system remained in place for 14 days to ensure thorough drainage and healing.

For non‐posterior space abscesses: In cases of superficial abscesses identified either during surgery or as a postoperative recurrence, a different approach was used. An indwelling needle was inserted, directly connected to the infusion extension tube and linked to the POVD system. This technique, as illustrated in Figure 2D, was particularly effective for managing shallower abscesses. The indwelling needle and POVD system were removed after 72 h.

**Figure 4:**
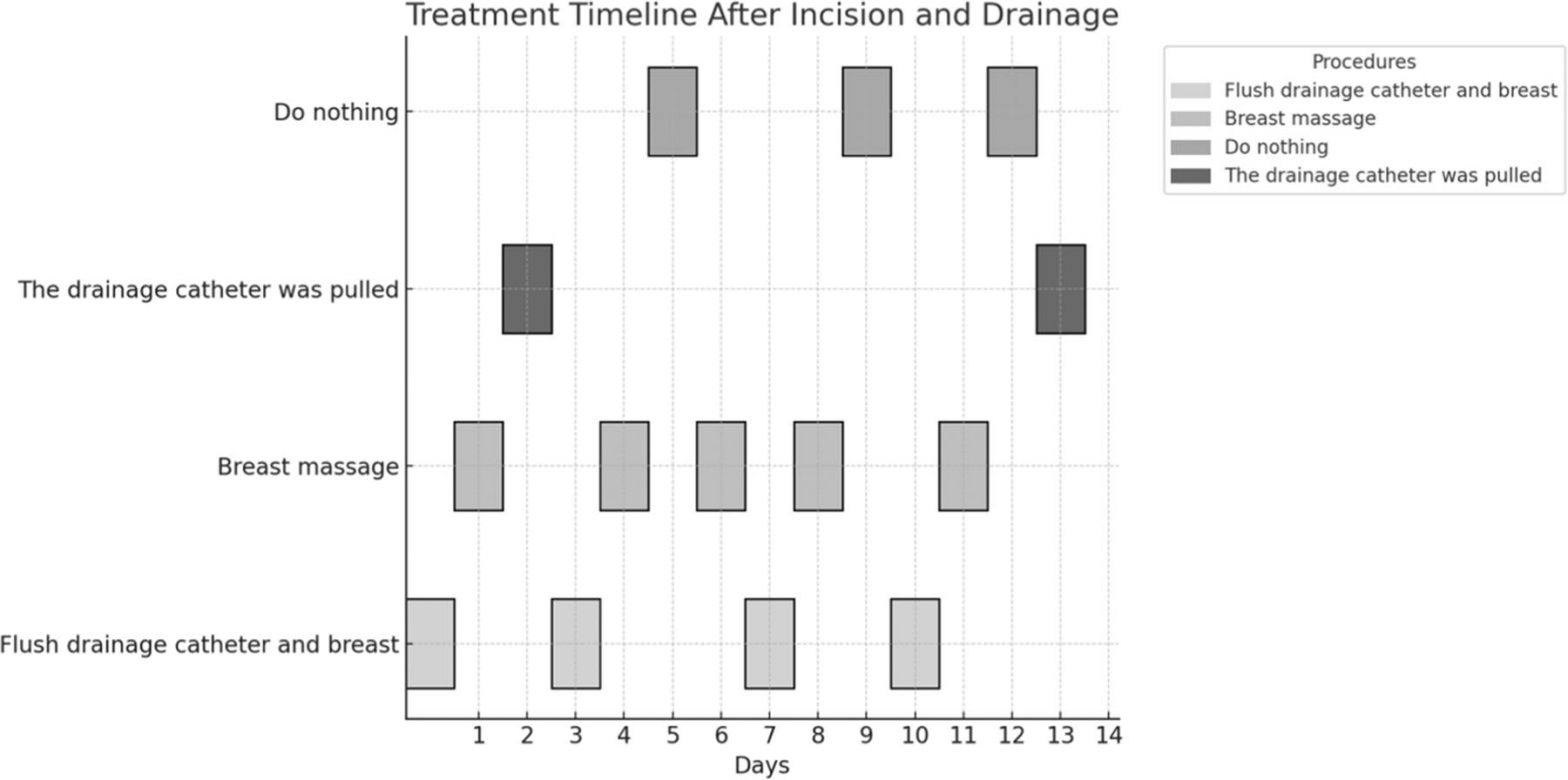
A schematic diagram illustrating the treatment protocol following incision and drainage is shown. On the day of surgery, a drainage catheter was inserted. The following day, the catheter was flushed, and gentle breast massage was initiated. Breast massage is particularly beneficial for patients with larger abscesses, especially deep breast abscesses, as it helps promote pus discharge and supports wound healing. The massage should always be performed gently and is part of a comprehensive treatment plan, rather than a standalone therapy. The entire procedure lasted for two weeks, ensuring optimal recovery while minimizing complications.

Postoperative Treatment Timeline: Figure 4 illustrates the Postoperative treatment timeline for patients undergoing incision and drainage. A drainage catheter was placed immediately after surgery to facilitate fluid removal. The timeline shows subsequent daily treatments over a 14‐day period, which included flushing the drainage catheter and performing breast massage to ensure proper fluid evacuation and reduce complications such as blockage or infection. The frequency of these treatments was alternated every 2–3 days. On day 13, the drainage catheter was removed, marking a key step in the recovery process.

Postoperative Follow‐Up: Patients were followed up with subsequent clinic visits. In cases where abscess remission was followed by recurrence, repeated POVD procedures were performed as necessary.

### 2.3 Pain Assessment

The Visual Analog Scale (VAS) was used to assess pain levels both before and after the operation. The scale consists of a line with no marks, numbers, or words, where one end represents “no pain” and the other represents “worst pain.” Patients were asked to mark a point on the line that best represented their level of pain. Pain levels were estimated based on the patient's position on the scale. The VAS range is from 0 (no pain) to 10 (worst pain), with higher scores indicating more severe pain.

### 2.4 Complete Response

Complete response was defined as meeting all of the following criteria:VAS ≤ 1; Complete resolution of local symptoms, such as skin erythema, pain, and swelling; Disappearance of fistula (if present); The patient's ability to return to normal daily activities without medical assistance; Disappearance of any palpable mass.

### 2.5 Statistical Analyses

Data processing and statistical analyses were performed using the SPSSAU data scientific analysis platform (https://spssau.com/). Continuous and ordinal variables, including age and VAS scores, were non‐normally distributed and were therefore expressed as median (interquartile range, IQR) and compared using the Mann‐Whitney U test. Categorical variables were compared using the chi‐square test or Fisher's exact test, as appropriate.

For the analysis of VAS score reduction (ΔVAS), ΔVAS was categorized into low (≤ 1), moderate (2–3), and high (≥ 4) pain reduction due to its ordinal and non‐normal distribution. An ordinal logistic regression model was fitted to assess the association between ΔVAS categories and treatment modality (combined operations vs. percutaneous aspiration), adjusting for age, BMI, diabetes, steroid use, abscess location (posterior vs. non‐posterior), IGM status, and abscess size. Odds ratios (OR), 95% confidence intervals (CI), and *p*‐values were reported. The proportional odds assumption was tested using the Brant test, and multicollinearity was assessed using variance inflation factors (VIF). All analyses were performed using SPSSAU (https://spssau.com/), with a significance threshold of *p* < 0.05.

From an initial 206 patients screened, 98 were included in the final cohort. A prior power analysis, targeting a power of 0.80 at a 0.05 significance level, validated this sample size. All statistical tests were two‐tailed with a significance threshold set at *p* < 0.05.

## 3 Result

### 3.1 Baseline Features

Our study screened a total of 206 patients, of whom 98 were included in the final analysis cohort (Figure 1). All participants were female, aged between 18 and 45 years (M ± SD; 30.5 ± 8.6). The mean BMI was 22.8 (SD = 3.7) kg/m², and the average abscess size was 4.2 (SD = 2.0) cm. Seventy‐seven patients were diagnosed through percutaneous needle biopsy, while 21 were confirmed via surgical biopsy. The final diagnoses included 59 cases of plasmacytic mastitis, 31 cases of granulomatous mastitis, and the remaining cases were nonspecific types of inflammation. During the 2‐year follow‐up period, one patient relapsed at 13 months, and one patient developed bilateral mastitis at 5 months.

The baseline characteristics of the two patient groups, including abscess size, smoking status, BMI, age, diabetes prevalence, history of immunosuppressive therapy, corticosteroid use, parity, trauma history, and treatment modality, were compared (Table 1). There were no statistically significant differences between the groups (*p* > 0.05).

**Table 1:** Baseline characteristics of patients' features.

**Species**	**Posterior space abscess group**	**Non‐posterior space abscess group**	**t/u/χ2**	***p*‐value**
Abscess size (cm)			0.67	0.540
≥ 5	18	27		
< 5	22	31		
Abscess			1.502	0.220
Single	13	16		
Multiple	27	32		
Smoking	4	6	0.003	0.956
Age (years), median (IQR)	28 (23–31)	30 (26–33)	1008.5	0.222
Diabetes (%)	5 (12.5%)	4 (6.8%)	0.38	0.538
Immunosuppression (%)	2 (5.0%)	0 (0.0%)	1.01	0.314
Steroid Use (%)	22 (55.0%)	29 (49.2%)	0.13	0.714
BMI(kg/m^2^)			0.421	0.675
≤ 18.5	1	4		
18.6–24.9	31	42		
> 24.9	8	12		
Parity			0.967	0.326
No	17	19		
Yes	23	39		
Interval since lactation			0.376	0.708
≤ 1 y	3	4		
1–5 y	15	26		
≥ 5 y	5	9		
Unilateral or bilateral			0.001	0.970
Unilateral	38	55		
Bilateral	2	3		
Trauma history	7	13	0.284	0.594
Modality of treatment			0.335	0.563
Percutaneous aspiration	21	27		
Combined operations	19	31		
Total	40	58		

Abbreviation: BMI, body mass index.

### 3.2 Treatment Outcomes in Two Groups

The preoperative VAS scores in the posterior space abscess group and the non‐posterior space abscess group were 7 [IQR: 5–8] and 5 [IQR: 3–6], respectively (*U* = 1615.0, *p* = 0.002). Severe pain (VAS ≥ 8) was reported by 13 patients in the posterior group and 7 in the non‐posterior group. Postoperative VAS scores were 4 [IQR: 2–5] in both groups (*U* = 1241.0, *p* = 0.661), and no patients in the posterior group reported severe pain after treatment, while one patient in the non‐posterior group did.

The complete response time in the posterior space abscess group and the non‐posterior space abscess group was 6.0 (SD = 1.9) and 5.0 (SD = 2.0) months, respectively (*p* < 0.05). One patient in the posterior space abscess group had a complete response time of ≥ 12 months. This patient, who had recurrent ipsilateral breast abscesses, was diagnosed with significant hyperprolactinemia (serum prolactin 803 ng/ml) and showed gradual improvement after 6 months of treatment with bromocriptine.

Total costs significantly differed between the two groups (*p* < 0.05), while postoperative VAS scores and puncture times did not significantly differ between the groups (*p* > 0.05) (Table 2).

**Table 2:** Comparison of treatment outcomes between the two groups.

Species	Posterior space abscess group	Non‐posterior space abscess group	t/χ2	*p*‐value
Complete response time			1.998	0.050
≤ 5 m	19	33		
6–11 m	20	17		
≥ 12 m	1	0		
VAS scores before operation			3.442	0.001
≤ 3	5	15		
4–7	22	37		
≥ 8	13	6		
VAS scores after operation			0.498	0.620
≤ 3	15	23		
4–7	25	35		
≥ 8	0	0		
Puncture times	3.8 ± 1.5	3.4 ± 1.3	1.385	0.169
Total costs (USD)	730 ± 173	520 ± 280	4.519	0.001
Total	40	58		

*Note:* Complete response time: The time required for full symptom resolution and abscess healing.

Abbreviation: VAS, visual analog scale.

**Figure 5:**
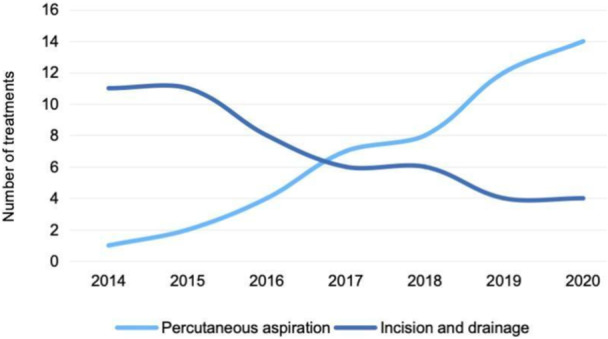
Trend of two treatment modalities (percutaneous aspiration vs. incision and drainage) over the 7‐year period from 2014 to 2020.

From 2014 to 2020, the number of patients undergoing incision and drainage treatment decreased by 69.4% (from 11 procedures in 2014 to 4 in 2020), while the number of patients receiving percutaneous aspiration treatment increased each year (Figure 5).

### 3.3 Regression Analysis of VAS Score Reduction

An ordinal logistic regression model was used to evaluate the change in VAS scores (ΔVAS), categorized as low (≤ 1), moderate (2–3), or high (≥ 4) pain reduction (Table 5). After adjusting for age, BMI, diabetes, steroid use, abscess location, IGM status, and abscess size, combined operations were associated with significantly greater pain reduction compared to percutaneous aspiration (OR = 2.85, 95% CI: 1.32–6.15, *p* = 0.008). Patients with IGM showed a trend toward greater pain reduction (OR = 2.12, 95% CI: 0.92–4.88, *p* = 0.077), though this was not statistically significant. Abscess location (posterior vs. non‐posterior), age, BMI, diabetes, steroid use, and abscess size were not significantly associated with ΔVAS (all *p* > 0.05). The model showed acceptable fit (pseudo‐*R*² = 0.152, *p* = 0.002), and the proportional odds assumption was not violated (*p* > 0.05) Table 3.

**Table 3:** Ordinal logistic regression analysis of VAS score reduction (ΔVAS).

Variable	β	SEx	*z*	Wald χ²	*p*‐value	OR	95% CI
Treatment Modality (Combined vs. Aspiration)	1.047	0.392	2.671	7.134	0.008	2.85	1.32–6.15
Abscess Location (Posterior vs. Non‐Posterior)	0.483	0.392	1.232	1.518	0.219	1.62	0.75–3.50
Age (years)	−0.020	0.027	−0.741	0.549	0.458	0.98	0.94–1.03
BMI (kg/m²)	0.039	0.048	0.822	0.676	0.412	1.04	0.94–1.15
Diabetes (Yes vs. No)	0.113	0.657	0.172	0.030	0.865	1.12	0.31–4.09
Steroid Use (Yes vs. No)	−0.274	0.386	−0.710	0.504	0.479	0.76	0.36–1.62
IGM Status (IGM vs. Non‐IGM)	0.751	0.425	1.767	3.123	0.077	2.12	0.92–4.88
Abscess Size (cm)	0.077	0.083	0.927	0.859	0.355	1.08	0.92–1.27

### 3.4 Two Treatment Modalities in Posterior Space Abscess Group

In the posterior space abscess group, the treatment outcomes of combined operations were significantly better than those of percutaneous aspiration. Specifically, in the posterior space abscess group, 20 patients received percutaneous aspiration and 18 underwent combined surgery. The preoperative VAS score in the aspiration group was 6 [IQR: 3–7], and the postoperative VAS score was 4 [IQR: 2–5], resulting in a VAS reduction of 1 [IQR: 0–2]. In comparison, the combined group had a higher baseline VAS score of 7 [IQR: 7–8], with a postoperative score of 4 [IQR: 1–5], and a greater VAS reduction of 3 [IQR: 2–4]. The difference in pain reduction between the two groups was statistically significant (*U* = 89.5, *p* = 0.007), indicating superior pain relief with combined surgery despite the higher initial pain level.

**Figure 6:**
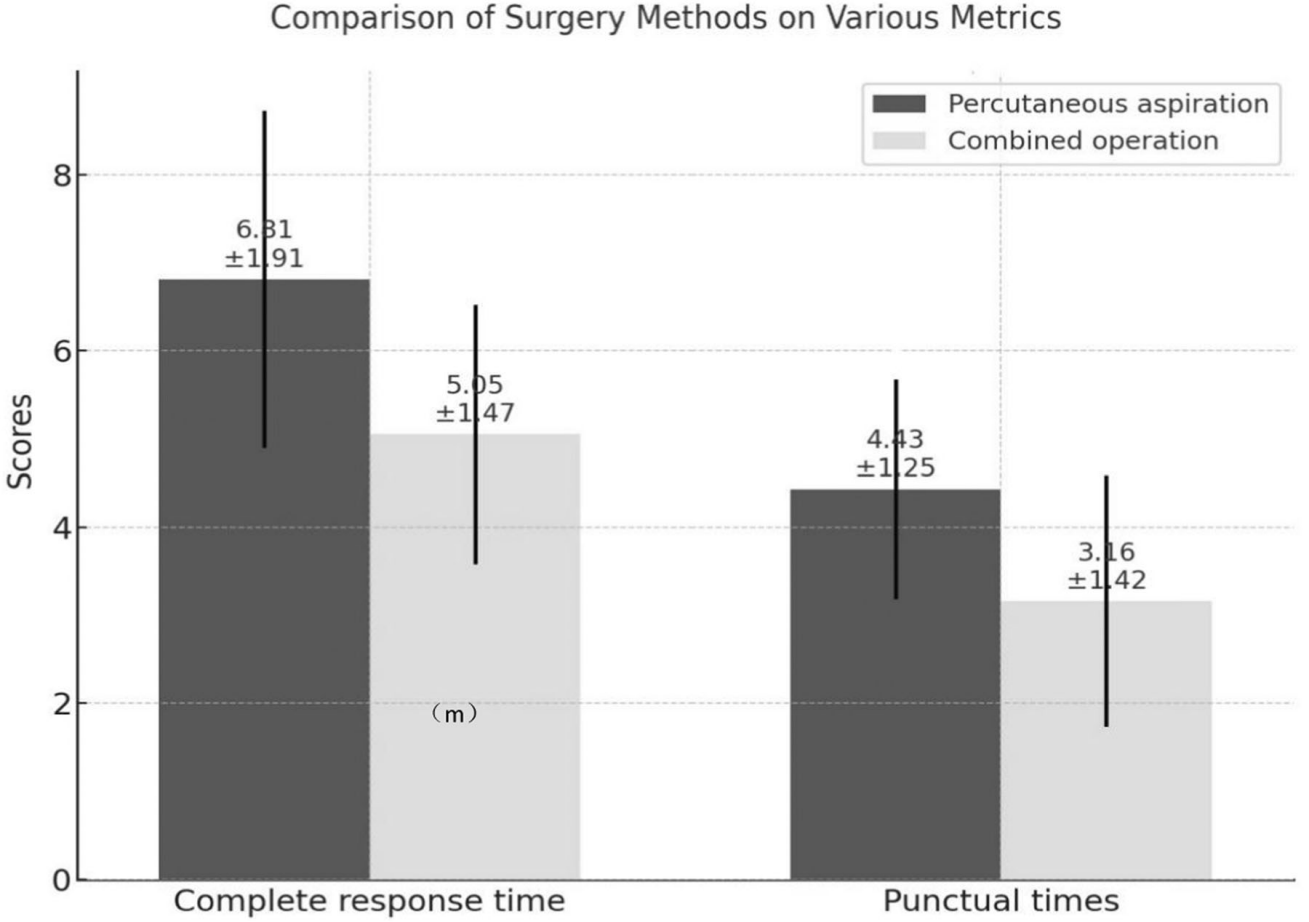
Comparison of two treatment modalities (percutaneous aspiration vs. combined operations) in posterior space abscess group.

**Figure 7:**
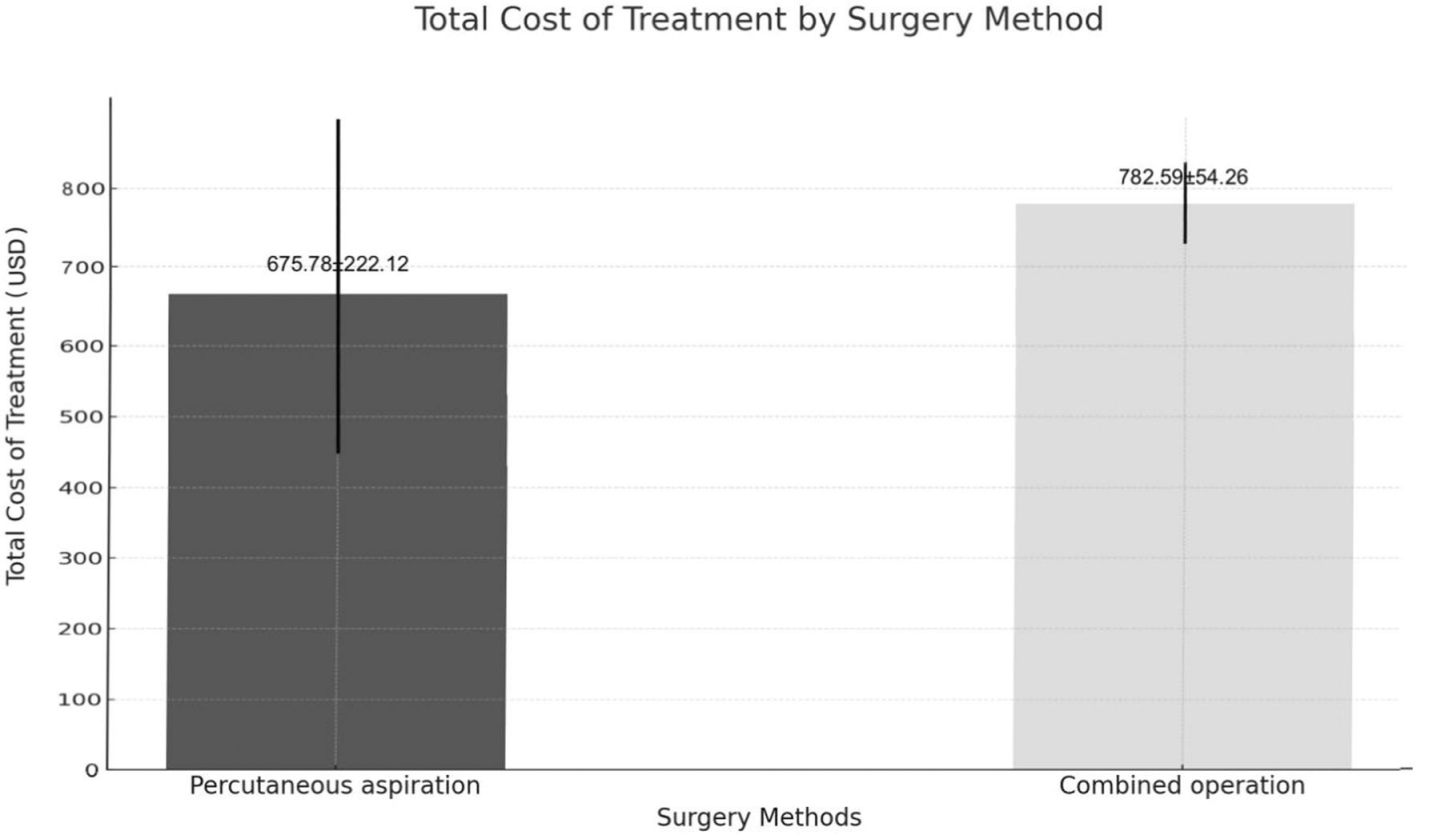
Total costs of two treatment modalities (percutaneous aspiration vs. combined operations).

Additionally, combined operations resulted in shorter complete response times and fewer puncture procedures compared to percutaneous aspiration. However, the total costs of combined operations were higher than those of percutaneous aspiration (*p* < 0.05) (Figure 6 and Figure 7).

### 3.5 Subgroup Analysis of Idiopathic Granulomatous Mastitis (IGM) Patients

Among the 98 patients, 31 (31.6%) were diagnosed with idiopathic granulomatous mastitis (IGM). Compared to the non‐IGM group (*n* = 67), IGM patients exhibited significantly higher preoperative pain (VAS: 8 [IQR: 7–8] vs. 5 [IQR: 3–6], *U* = 1918.0, *p* < 0.001), higher postoperative pain (VAS: 6 [IQR: 4–7] vs. 4 [IQR: 2–4], *U* = 1509.0, *p* < 0.001), and longer complete response time (6 months [IQR: 5–8] vs. 5 months [IQR: 4–6], *U* = 1423.5, *p* = 0.003).

The recurrence rate was also significantly higher in the IGM group (16.1% vs. 3.0%, χ² = 5.412, *p* = 0.020). No significant differences were observed in age, BMI, bilateral involvement, or the incidence of multiple abscesses (all *p* > 0.05). These results highlight the more severe and refractory nature of IGM. (Table 4)

**Table 4:** Comparison of clinical characteristics between IGM and Non‐IGM Groups.

Variable	IGM (*n* = 31)	Non‐IGM (*n* = 67)	U/χ2	*p*‐value
Age (years), median (IQR)	35 (26–41)	28 (23–36)	1247.5	0.111
BMI (kg/m²), median (IQR)	21.7 (20.0–24.1)	21.7 (20.0–24.7)	1027.0	0.933
Pre‐op VAS, median (IQR)	8 (7–8)	5 (3–6)	1918.0	< 0.001
Post‐op VAS, median (IQR)	6 (4–7)	4 (2–4)	1509.0	< 0.001
Complete response time, months	6 (5–8)	5 (4–6)	1423.5	0.003
Recurrence rate, %	16.1% (5/31)	3.0% (2/67)	5.412	0.020
Bilateral involvement, %	12.9% (4/31)	9.0% (6/67)	0.489	0.484
Multiple abscesses, %	58.1% (18/31)	55.2% (37/67)	0.257	0.612

Among IGM patients (*n* = 31), no significant differences were observed between those treated with aspiration (*n* = 15) and combined surgery (*n* = 16) in terms of postoperative pain (median VAS: 6 vs. 6, *p* = 0.699) or complete response time (both median 6 months, *p* = 0.904). Although the combined group showed a trend toward greater VAS Score Reductions (median ΔVAS: 2 vs. 1), the difference was not statistically significant (*p* = 0.155), suggesting comparable efficacy between the two approaches (Table 5).

**Table 5:** Comparison of clinical outcomes between aspiration and combined surgery in IGM patients.

Outcome	Apercutaneous aspiration (*n* = 15)	Combined Operation (*n* = 16)	*U*	*p*‐value
VAS improvement (ΔVAS)	1 (1–2)	2 (1–4)	85.0	0.155
Postoperative VAS	6 (4–7)	6 (4–7)	130.0	0.699
Complete response time (months)	6 (5–8)	6 (5–8)	123.5	0.904

## 4 Discussion

This study demonstrated that posterior space abscesses in non‐lactational mastitis are associated with more severe symptoms, including higher pain levels and longer recovery times compared to non‐posterior abscesses. This contrasts with non‐posterior space abscesses, which presented less severe symptoms and required different treatment approaches. This distinction between posterior and non‐posterior space abscesses was made to address the clinical challenges posed by the deeper location and more severe symptoms of posterior space abscesses. Unlike non‐posterior abscesses, which are more accessible and manageable with minimally invasive methods such as ultrasound‐guided aspiration, posterior space abscesses often require a combination of incision, drainage, and vacuum‐assisted drainage to achieve optimal outcomes. This classification is particularly significant because it enables clinicians to tailor treatment strategies based on abscess location, improving patient outcomes while reducing unnecessary interventions. By providing a framework for location‐specific treatment approaches, this study pioneers a novel perspective in the management of non‐lactational mastitis, laying the foundation for further research in this area.

Vacuum‐assisted drainage systems, such as Postoperative Vacuum Drainage (POVD), have been successfully applied in other medical fields, such as wound management, plastic surgery, and orthopedics. These systems are proven effective in enhancing wound healing, reducing infection rates, and minimizing hospital stays. However, their use in non‐lactational mastitis, particularly for deep‐seated posterior space abscesses, is relatively new and less explored. Our study demonstrates that while the combined treatment approach—integrating POVD with incision and drainage—slightly increases costs, primarily due to the incision and drainage procedure, it significantly improves pain relief, shortens recovery time, and reduces the number of interventions compared to percutaneous aspiration alone. To further quantify the effectiveness of combined operations, an ordinal logistic regression analysis was conducted, incorporating regression coefficients, standard errors, and Wald statistics (Table 5). This analysis confirmed the superiority of combined operations over percutaneous aspiration for pain reduction (β = 1.047, OR = 2.85, 95% CI: 1.32–6.15, *p* = 0.008), with a significant Wald χ² (7.134) underscoring the robustness of this finding. This supports the observed advantage of combined operations, particularly for posterior space abscesses, which are associated with higher baseline pain levels. A trend toward greater pain reduction in patients with idiopathic granulomatous mastitis (IGM) (β = 0.751, OR = 2.12, 95% CI: 0.92–4.88, *p* = 0.077) suggests that immune‐mediated pathology may influence treatment response, warranting further investigation. Nonsignificant covariates, such as abscess location (*p* = 0.219) and diabetes (*p* = 0.865), indicate that treatment modality is the primary driver of pain reduction outcomes in this cohort. These findings highlight the clinical value of combined operations in achieving meaningful pain relief, enhancing patient recovery.

Our results align with previous studies on non‐lactational mastitis treatments, yet highlight important distinctions. For example, Irusen et al. [11] and Naeem et al. [16] demonstrated the benefits of ultrasound‐guided needle aspiration for lactational abscesses, showing faster recovery compared to traditional incision and drainage (I&D). Similarly, Christensen et al. [17] reported that ultrasound‐guided drainage reduced the need for invasive surgery, consistent with our findings regarding the effectiveness of minimally invasive techniques. Wang et al. [18], Chandika et al. [19], and Ozseker et al. [20] emphasized the advantages of minimally invasive approaches for managing breast abscesses, including needle aspiration and ultrasound‐guided drainage, which improved clinical outcomes in various settings. However, unlike these studies, which primarily employ minimally invasive methods for all abscesses, our research distinguishes between posterior and non‐posterior space abscesses. We found that combined operations are more suitable for posterior space abscesses, highlighting their unique characteristics and underscoring the differences in treatment approaches for these two types of abscesses.

Building on the regression analysis suggesting a trend toward greater pain reduction in IGM patients (Table 3, OR = 2.12, *p* = 0.077), the subgroup analysis of IGM patients (*n* = 31) revealed no significant differences in postoperative pain or complete response time between combined operations and percutaneous aspiration (Table 5, *p* > 0.05), despite a trend toward greater VAS reduction with combined operations (median ΔVAS: 2 vs. 1, *p* = 0.155). This similarity in outcomes may be attributed to IGM's immune‐mediated etiology, which involves chronic granulomatous inflammation driven by autoimmune or hypersensitivity responses [3, 6]. Unlike other non‐lactational mastitis subtypes, IGM may require adjunctive immunosuppressive therapies, such as corticosteroids or methotrexate, to address the underlying immune dysregulation, rather than relying solely on surgical or drainage interventions [3, 8]. The lack of significant superiority of combined operations in IGM patients suggests that POVD, while effective for drainage, may not fully address the inflammatory pathology of IGM. Further studies are needed to evaluate the role of combined surgical and immunosuppressive strategies in optimizing IGM treatment outcomes.

Previous studies [21, 22] have confirmed that vacuum‐assisted drainage systems (negative pressure) significantly enhance wound healing by promoting granulation tissue formation, reducing infection rates, and improving overall healing outcomes. This approach has proven particularly effective in various clinical settings, accelerating recovery and minimizing complications. However, there is still a lack of large studies describing its effectiveness in breast surgery [23]. Chen et al. [13] and Wang et al. [18] support the efficacy of comprehensive minimally invasive treatments like vacuum‐assisted drainage. However, these studies have not distinguished between posterior and non‐posterior space abscesses in their application of negative pressure drainage methods. In our approach, we use a simplified negative pressure drainage device (POVD) after incisional drainage for posterior space abscesses, while for non‐posterior space abscesses, we employ an indwelling needle connected to the POVD under ultrasound guidance. Unlike traditional vacuum‐assisted devices, our POVD does not require specialized equipment; it creates negative pressure using a syringe, with adjustable suction strength. This device is user‐friendly, highly accessible, and cost‐effective, making it particularly suited for patients in underdeveloped or resource‐limited settings. These advantages make POVD a practical and adaptable solution for non‐lactational mastitis, extending the accessibility of effective treatment [15, 18, 24]. Previous studies suggest that the incidence of non‐lactational mastitis is increasing, particularly in urban coastal regions, although there is considerable debate about the optimal treatment strategies. For instance, Bouton et al. [25] questioned the necessity of traditional surgical incision and drainage for non‐lactational mastitis. Our research contributes to this discussion by distinguishing between posterior and non‐posterior space abscesses. We found that non‐posterior space abscesses often present with milder symptoms, allowing effective treatment through ultrasound‐guided aspiration combined with POVD, potentially eliminating the need for surgical intervention. In contrast, posterior space abscesses are associated with more severe symptoms, emphasizing the need for surgical incision. However, unlike conventional approaches, our treatment protocol incorporates a combined method that includes POVD, demonstrating its efficacy. This nuanced approach helps address ongoing debates about when incision and drainage are necessary and contributes to the evolving perspective on the surgical management of non‐lactational mastitis.

One of the hypothesized mechanisms underlying the improved outcomes in our study is the tailored application of treatment modalities based on abscess location. For posterior space abscesses, which are typically deeper and more difficult to treat, the combined approach appears to more effectively address the severity of these abscesses [26]. The inclusion of POVD offers several advantages: its minimally invasive nature reduces patient discomfort, its adjustable pressure settings allow for more controlled drainage, and its ease of use improves patient compliance while reducing the burden on healthcare providers. These findings suggest the need for a more nuanced approach to assessing breast abscess cases. Rather than using a uniform treatment method, clinicians should evaluate abscess characteristics such as location, size, and severity to guide treatment decisions. This personalized approach could lead to better patient outcomes, reduced treatment times, and potentially lower healthcare costs.

Our study has several limitations that must be considered. The retrospective design of this study did not involve patient matching, which may have introduced selection bias. However, the comparison of baseline characteristics showed no significant differences between the two groups. The small, homogeneous sample size may limit the generalizability of the findings, making it difficult to apply the results to broader populations. The absence of randomized controlled trials (RCTs) reduces the robustness of the evidence compared to studies that include RCTs. Additionally, the lack of direct comparisons with studies using different methodologies or treatment approaches leaves our findings somewhat isolated and highlights the need for further research to validate the results. For example, the exclusion of a conservative observation group from the control cohort may have hindered our ability to clearly differentiate outcomes between conservative treatment and surgical intervention, which we intend to explore in future clinical studies. Furthermore, the study does not include a comprehensive cost‐effectiveness analysis or long‐term data on the efficacy and safety of the combined treatment approach, which are essential for evaluating its broader applicability and sustainability. Finally, the standalone effectiveness of POVD could not be assessed, as it was consistently used as part of combined operations or following aspiration in our protocol. Future studies should investigate POVD as an independent intervention to elucidate its individual contribution to treatment outcomes.

Our study introduces a significant advancement in the treatment of non‐lactational mastitis, particularly in the management of posterior space abscesses, by utilizing a novel combined treatment approach incorporating POVD. This approach, which emphasizes the importance of distinguishing between abscess locations, provides a minimally invasive, cost‐effective method that improves patient outcomes. Further research, including larger‐scale studies and long‐term follow‐up, is needed to confirm the broader applicability and long‐term benefits of this approach.

## Author Contributions

**WenJie Zhang:** formal analysis, investigation, methodology, writing – original draft. **Jian Wu:** conceptualization, writing – review and editing, **Hailiang Ren:** data curation,softwarecuration, software.

## Ethics Statement

This study was conducted in accordance with the ethical standards of the institutional and national research committee and with the 1964 Helsinki Declaration and its later amendments or comparable ethical standards. All procedures performed in this study involving human participants were in line with the ethical standards of the institutional research committee at the Third Hospital of Chengdu and the national research council.

Informed consent was obtained from all individual participants involved in the study. Participant data were deidentified and confidentiality was ensured throughout the study and subsequent data analysis process. This study did not involve any animals.

## Conflicts of Interest

The authors declare no conflicts of interest.

## Data Availability

The datasets used and/or analysed during the current study available from the corresponding author on reasonable request.
